# Validity and reliability of the Spanish version of the Organizational Readiness for Knowledge Translation (OR4KT) questionnaire

**DOI:** 10.1186/s13012-017-0664-y

**Published:** 2017-11-10

**Authors:** Gonzalo Grandes, Paola Bully, Catalina Martinez, Marie-Pierre Gagnon

**Affiliations:** 1 Primary Care Research Unit of Bizkaia, Basque Health Service-Osakidetza, Luis Power 18, 4a planta, E-48014 Bilbao, Spain; 2 BioCruces Health Research Institute, Plaza de Cruces 12, E-48903 Barakaldo, Bizkaia Spain; 3 Population Health and Optimal Health Practices Research Unit, CHU de Québec-Université Laval Research Centre, QC, Québec G1V 0A6 Canada; 4 Faculty of Nursing, Université Laval, 2325 Rue de l’Université, Ville de Québec, QC G1V 0A6 Canada

**Keywords:** Knowledge translation, Implementation, Organizational readiness to change, Healthy lifestyle, OR4KT, Psychometric properties, Primary health care

## Abstract

**Background:**

Organizational readiness to change healthcare practice is a major determinant of successful implementation of evidence-based interventions. However, we lack of comprehensive, valid, and reliable instruments to measure it. We assessed the validity and reliability of the Spanish version of the Organizational Readiness for Knowledge Translation (OR4KT) questionnaire in the context of the implementation of the *Prescribe Vida Saludable III* project, which seeks to strengthen health promotion and chronic disease prevention in primary healthcare organizations of the Osakidetza (Basque Health Service, Spain).

**Methods:**

A cross-sectional study was conducted including 127 professionals from 20 primary care centers within Osakidetza. They filled in the OR4KT questionnaire twice in a 15- to 30-day period to test repeatability. In addition, we used the Survey of Organizational Attributes for Primary Care (SOAPC) and we documented the number of healthcare professionals who formally engaged in the *Prescribe Vida Saludable III* project within each participating center to assess concurrent validity.

**Results:**

Cronbach’s alpha for the overall OR4KT was .95, and the overall repeatability coefficient was 6.95%, both excellent results. Confirmatory factor analysis supported the underlying theoretical structure of 6 dimensions and 23 sub-dimensions. There were positive moderate-to-high internal correlations between these six dimensions, and there was evidence of good concurrent validity (correlation coefficient of .76 with SOAPC, and .80 with the proportion of professionals engaged by center). A score higher than 64 (out of 100) would be indicative of an organization with high level of readiness to implement the intervention (sensitivity = .75, specificity = 1).

**Conclusions:**

The Spanish version of the OR4KT exhibits very strong reliability and good validity, although it needs to be validated in a larger sample and in different implementation contexts.

**Electronic supplementary material:**

The online version of this article (10.1186/s13012-017-0664-y) contains supplementary material, which is available to authorized users.

## Background

The Organizational Readiness for Knowledge Translation (OR4KT) questionnaire was developed to assess healthcare organizations’ readiness to change clinical practice in order to adopt proven interventions [1]. Implementing evidence-informed practice represents an ongoing challenge [2]. In this paper, we focus on health promotion in primary health care, an excellent example of the huge gap between knowledge and practice. Despite the sound epidemiological evidence for the impact of individual behavior on population health [3], the success of implementing interventions for lifestyle promotion in routine primary care has been limited to date [4]. It is generally accepted that despite their good knowledge and positive attitudes towards health promotion, primary healthcare providers face a range of organizational and contextual barriers to providing lifestyle interventions [5–8]. In this context, assessing primary care organizations’ level of readiness for lifestyle promotion interventions could help us improve the chances that these practices are successfully adopted.

Several instruments have been developed to assess organizational readiness to change [9–11], but few of them specifically concern the implementation of evidence-informed health promotion interventions in primary care. For instance, the Organizational Readiness for Implementing Change (ORIC) was developed by Shea et al. [12] drawing on Weiner’s theory of organizational readiness for change [11]. Authors assessed its content adequacy, structural validity, reliability, and construct validity in a series of studies. Although the ORIC shows promise, more research is needed to test for convergent, discriminant, and predictive validity and assess its performance in a primary care setting [12].

Further, the Assessment of Readiness for Chronicity in Health Care Organizations (IEMAC from the Spanish, *Instrumento de Evaluación de Modelos de Atención ante la Cronicidad*) is a measurement tool specifically designed to assess readiness to implement the conceptual framework of the Chronic Care Model in the Spanish national health system [13]. This instrument allows healthcare organizations to perform self-assessments regarding their readiness to provide integrated care for coping with chronicity and to identify areas for improvement in chronic care. Mira et al. [14] assessed the preliminary validity and reliability of the IEMAC. Although this instrument presents fair psychometric properties, it has not been designed to assess organizational readiness to implement a specific intervention.

Weeks et al. [15] developed the Diabetes Care Coordination Readiness Assessment (DCCRA) to assess primary care clinic readiness to coordinate care for adult patients with diabetes. This self-administered tool can be used for informing primary care organizations of their readiness for care coordination and identifying gaps in current practices. It can also be administered over time to assess progress in care coordination capacity, inform program evaluation, and assist quality improvement initiatives. Although the DCCRA could potentially be used to assess readiness for care coordination in other chronic conditions seen in primary care, it has not been adapted for wider use.

Recently, the OR4KT has been proposed as a generic instrument to assess the readiness of healthcare organizations for change to enable the implementation of evidence-informed practice in the field of chronic care [1]. In the development of the OR4KT, organizational readiness for change was considered as a multidimensional collective construct, covering both the psychological (i.e., motivational) aspects of the members of an organization and the structural factors related to human and technical resources, as specified in the protocol [16]. It combines generic scales adapted from existing validated ORC instruments that can be tailored to specific changes depending on the organizational context [17]. Thus, the OR4KT operationalizes concepts for the assessment of healthcare organization capacities to engage in any kind of evidence-based change. Its main focus is on the implementation of change, but we hypothesize that the OR4KT could also be useful for monitoring change progression in an organization.

This tool was developed in English and has been translated into French and Spanish and cross-culturally adapted for use in Canada and Spain. The aim of the present study was to assess the validity and reliability of the Spanish version of the OR4KT in the context of the implementation of evidence-based interventions for health promotion and chronic disease prevention in primary healthcare organizations in the Basque country. Specifically, the OR4KT was used in the Prescribe Healthy Life III (PVS-III, an abbreviation from the Spanish: *Prescribe Vida Saludable III*) project, whose objective was to optimize health promotion practice in primary care through implementation research [5–7, 18, 19]. The implementation strategy used in PVS-III relies on discussion and consensus meetings for needs assessment and bottom-up decision making. Accordingly, this strategy requires the commitment of the majority of the professionals from highly motivated primary care centers. The OR4KT was used to identify primary care centers ready to initiate this practice-changing process.

## Methods

### Design

We conducted a cross-sectional study with repeated measures in three integrated healthcare organizations of the Basque Health Service (*Osakidetza*) in order to validate and assess the reliability of the Spanish version of the OR4KT questionnaire.

### Development of the OR4KT questionnaire

The OR4KT was developed following a three-phase process. In the initial development phase, we first conducted systematic reviews of the theoretical underpinnings of organizational readiness (OR) and existing OR measurement instruments to provide a pool of relevant conceptual dimensions and related items [17, 20]. Then, we conducted a modified two-round Delphi survey among international experts to assess content validity of the pilot OR4KT instrument [21]. In the second development phase, the preliminary OR4KT instrument (containing 91 items) went through an item-reduction process, involving a panel of seven international experts, seeking to decrease its length. The final OR4KT questionnaire includes 59 items assessing 6 dimensions and 23 sub-dimensions related to the concept of organizational readiness. The third development phase was the translation of the pilot OR4KT instrument, originally developed in English, into French and Spanish and its cross-cultural adaption for use in Canada and Spain [1].

### Setting and participants

Convenience cluster sampling was carried out to select participants among the staff of 49 primary care units (*Unidades de Atención Primaria—UAP*) belonging to three integrated care organizations of Osakidetza. We sent a letter to the heads of these primary care units inviting their organization to participate in the project and we received a positive response from 20 of these units. Seven key informants from each unit were identified using snowball sampling, obtaining a total of 140 individual participants.

The first participant was the head of the unit, who had the official management responsibility for the unit staff, and he or she was asked to recruit one representative of each of the following groups: physicians, nurses, and administrative staff. Each of these representatives was then asked to recruit another individual among colleagues from their professional category (physician, nurse, or administrative staff) who they believed represented an average position in terms of their attitude towards health promotion, that is, neither very willing nor very reluctant.

### Data collection

The survey was composed of the Spanish version of the OR4KT, a culturally adapted Spanish version of the Survey of Organizational Attributes for Primary Care (SOAPC) [22], and characteristics of the primary care unit, as well as questions concerning the sociodemographic and professional characteristics of respondents. We also documented the number of healthcare professionals who formally engaged as collaborators in the PVS-III project within each participating primary care unit.

The Spanish version of the OR4KT instrument (Additional file 1) comprises 59 items and assesses 6 dimensions and 23 sub-dimensions related to organizational predisposition to knowledge translation: organizational climate, organizational support, contextual factors, change content, leadership, and motivation. It uses a 5-point Likert scale and the total OR4KT score is computed by summing the scores on each item, with a maximum score of 295 points. This score is then normalized on a 0 to 100 scale to ease interpretation.

We created a translated and culturally Spanish adapted version of the SOAPC, a 21-item questionnaire that assesses four organizational attributes that are relevant to primary care from the perspective of physicians, nurses, and other staff. The four scales of the SOAPC are (1) communication: healthcare professionals’ capacity to work as a team and solve problems through discussion (4 items); (2) decision making: healthcare professionals’ participation in decision making (8 items); (3) stress/chaos: healthcare professionals’ workload and the management of tension in the center (6 items); and (4) history of change in the organization (3 items). The items are rated on a 5-point Likert scale, from 1=totally disagree to 5=totally agree. The scores obtained are also transformed to obtain a normalized score ranging from 0 to 100 to facilitate the interpretation of the results. This version of the SOAPC has been validated using an expert panel and factor analysis, which confirmed its four-factor structure. The internal consistency of the scales was satisfactory, with Cronbach’s alphas of 0.81 for communication, 0.88 for decision making, 0.85 for stress/chaos, and 0.73 for history of change.

As indicated above, the survey instrument also included ad hoc questions to collect data on respondents’ sociodemographic (age, sex, and level of education) and professional (profession, position, work experience, and time in current position) characteristics. We also collected information on the primary care units, namely number of registered patients, number of healthcare professionals, mean number of registered patients per family physician or pediatrician, and a socioeconomic deprivation index for the catchment area that combines variables related to employment (unemployment rate, manual and short-term employment) and education (educational attainment rate among young people and overall).

The survey was completed a first time and then repeated under similar circumstances after an interval of 15 to 30 days.

Finally, for each participating primary care unit, we estimated the level of commitment to the project by calculating the proportion of professionals who engaged as collaborators in the PVS-III project. To do so, we held a 2-h informative meeting at each of the 20 selected primary care units to explain the objectives and methods of the PVS-III project. After the meeting, we collected individual signed consent forms from attendees who agreed to participate and collaborate in the project. Then, we calculated the percentage of professionals who committed to the project in each unit as an indicator of their intention to change their clinical practice to integrate health promotion in routine care. Units were categorized as ready to change clinical practice if more than 50% of their staff gave written informed consent to collaborating in the PVS-III project.

### Data analysis

To assess the psychometric properties of the Spanish version of the OR4KT questionnaire, we used classical test theory. The reliability of the measurement was quantified in terms of consistency and repeatability. Cronbach’s α coefficients were calculated to determine the internal consistency of the scales. A value of α ≥ 0.7 was considered good. The repeatability was determined by calculating the Coefficient of Repeatability (CR) [23].

To assess the instrument validity quantitatively, we considered internal and external evidences [24]. Internal evidence of the relationships between elements of the test and its agreement with the underlying theoretical model was evaluated through a confirmatory factor analysis (CFA) by the maximum likelihood mean-variance (MLMV) adjusted method, which is robust to non-normal distributions. In a first step, we adjusted a model in which we specified the sub-dimension of membership for each of the items. In the next step, we carried out a second-order analysis, checking the fit of a model in which each sub-dimension was loaded in its reference dimension and these in turn in a global dimension that measures the organizational readiness. The degree of fit to the data for each model was evaluated by reference to the value of the chi-square/degree of freedom ratio and also to the incremental Comparative Fit Index (CFI) and the Standardized Root Mean Square Residual (SRMR). Models with $$ \raisebox{1ex}{${\chi}^2$}\!\left/ \!\raisebox{-1ex}{$ df$}\right. $$ ≤ 5, CFI ≥ .90 and SRMR ≤ .08 are considered acceptable [25]. As external evidences, we assessed concurrent validity by exploring the association of the OR4KT dimensions with (1) the dimensions measured by the SOAPC, which includes similar and related constructs, and hence, it was hypothesized that the relationship would be positive and (2) the proportion of professionals who individually signed the commitment to participate in PVS-III, with an expected positive relationship, as individual willingness would correlate positively with organizational readiness. To investigate these potential relationships, bivariate correlations were calculated using Spearman’s rank order correlation (rho). Finally, we analyzed the known-groups validity through receiver operating characteristic (ROC) curves constructed from the mean total OR4KT scores in the health centers in which more than 50% of the staff signed consent forms agreeing to collaborate in the PVS-III project and in those in which this requirement was not met.

We used SAS (v. 9.2, SAS Institute, Cary, NC, USA) and R (R Development Core Team, 2014) to perform all statistical analyses.

## Results

### Participants

#### Primary care units

Among the 49 primary care units that were eligible to participate in this study, 33 replied to our invitation (66%), and of these, 20 agreed to participate in the study (40% of the total). We found no statistically significant differences in structural or sociodemographic characteristics between participating and non-participating primary care units (*p* > 0.1, data not shown).

#### Professionals

All of the 20 participating primary care units recruited and provided contact information on seven key informants. Among these potential 140 individual participants, 127 (90.7%) completed the first questionnaire and 90 (70.9%) the second questionnaire. Table 1 summarizes the characteristics of individual participants.

**Table 1:** Characteristics of participants

	Number	Percent
Integrated care organization		
Bilbao-Basurto	43	33.86
Barrualde-Galdakao	40	31.50
Ezkerraldea-Enkarterri	44	34.65
Sex		
Male	99	77.95
Female	28	22.05
Age group, years		
30–39	7	5.56
40–49	43	34.13
50–59	68	53.97
60–65	8	6.35
Highest level of education		
Primary or secondary	10	7.87
Vocational or technical	13	10.24
University diploma or equivalent	42	33.07
University degree or equivalent	62	48.82
Position		
Head of the primary care unit	19	14.96
Medical director	19	14.96
Nursing director	17	13.39
Administrative director	19	14.96
Physician	16	12.60
Nurse	19	14.96
Administrative staff	18	14.17
Length of experience, years		
0–9	5	3.94
10–19	30	23.62
20–29	54	42.52
30–39	35	27.56
40–50	3	2.36
Time in current position, years		
0–9	74	58.27
10–19	31	24.41
20–29	21	16.54
30–39	1	0.79

### Descriptive statistics of the OR4KT questionnaire

Table 2 presents the descriptive and reliability statistics for the six dimensions of the OR4KT questionnaire and its total score.

**Table 2:** Descriptive and reliability statistics of the OR4KT questionnaire

Dimensions	Min.	Max.	% ceiling	M	SD	Sk	Kurt.	*α*	%CR
Climate for change	20.00	92.50	0.00	57.70	12.44	0.04	0.26	.79	12.09
Contextual factors	35.00	85.00	0.00	57.43	11.39	− 0.10	− 0.61	.79	13.33
Change content	36.11	94.40	0.00	63.34	11.73	− 0.26	− 0.31	.84	12.13
Leadership	20.00	100.00	0.79	57.12	12.71	0.15	1.01	.83	13.26
Organizational support	5.00	100.00	0.79	60.35	14.01	− 0.79	2.13	.92	13.18
Motivation	10.00	100.00	0.79	58.02	11.05	− 0.53	3.70	.81	12.92
Total score	28.98	87.36	0.00	59.10	9.98	− 0.18	0.54	.95	6.95

*Min.* minimum score, *Max.* maximum score, *% ceiling* percentage of participants with the maximum score of 100, *M* mean, *SD* standard deviation, *Sk.* skewness, *Kurt.* kurtosis, *α* Cronbach’s alpha, *%CR* coefficient of repeatability

These results show that the OR4KT scores are distributed without noticeable deviations from the normal curve and that there are no important ceiling effects.

### Reliability

Generally, Cronbach’s alpha coefficients show very good internal consistency with values between .79 and .92 for the six dimensions of the OR4KT questionnaire and .95 for the total score. The repeatability of the questionnaire is also high for each of the six dimensions, with CRs of between 12.09 and 13.33% and a CR of 6.95% for the total score.

### Validity

#### Internal validity: dimensionality

**Fig. 1:**
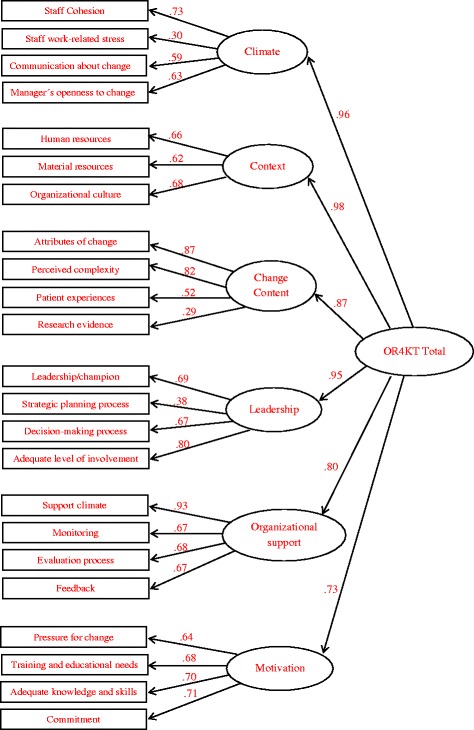
OR4KT CFA sub-dimensions and dimensions model

The configuration matrix (see Additional file 2) provides the saturation values of the items for each sub-dimension. The model shows an acceptable fit between the hypothesized theoretical model with 23 sub-dimensions and the empirical solution (*χ* ^2^⁄*df* = 1.75, CFI = .86, and SRMR = .08). The factor structure of the dimension composites was also analyzed using confirmatory factor analysis (Fig. 1). The results supported the existence of a second order structure beyond the sub-dimension composites (*χ* ^2^⁄*df* = 3.14, CFI = .84, and SRMR = .08).

The *Organizational climate for change* dimension considers the organizational dynamics that support cooperation and trust between staff. It includes the following sub-dimensions: staff cohesion (items 1 to 3), staff work-related stress (items 4 to 5), communication about change (items 6 to 8), and manager’s openness to change (items 9 and 10). However, the factor analysis showed that item 8 showed a statistically significant but low saturation in its sub-dimension.

The *Contextual factors* dimension comprises three sub-dimensions: human (item 11 and 15) and material resources (items 12 to 14) and organizational culture (items 16 to 20). These dimensions help us to understand the attributes of the organizational environment where the change takes place. However, item 15 does not fit well with the underlying factor.

The *Change content* dimension is formed by attributes of change (items 21 and 22), perceived complexity (items 23 to 25), patient experiences and preferences (items 26 to 28), and clinical evidence supporting changes (item 29). The performance of this last sub-dimension in the dimension is questionable.

The *Leadership/participation* dimension includes four sub-dimensions: leadership/champion (items 30 to 32), the strategic planning process (item 33), the decision-making process (items 34 to 36), and the adequate level of involvement (items 37 to 39). In this case, the loadings of items 32 and 33 are not optimal.

*Organizational support* includes four sub-dimensions: support climate (items 40 to 43), monitoring (items 44 and 45), evaluation process (items 46 to 48), and feedback (item 49). All the sub-dimension items showed good performance.

The *Motivation* dimension also theoretically covers four sub-dimensions: pressure for change (items 50 to 54), training and education needs (item 55), adequacy of knowledge and skills (items 56 and 57), and commitment (items 58 and 59). Empirically, the second-order structure is reproduced, but items 50, 52, and 53 have low loadings on their sub-dimension. Taken together, these results indicate a need to review if the problem is the underlying theoretical structure of this sub-dimension or the use of the term “pressure”.

#### Internal validity: relations between dimensions

Table 3 reports the Spearman’s correlation coefficients between the six dimensions of the OR4KT. As expected, the correlations between dimensions are all positive and statistically significant (*p* ≤ 0.001), with moderate-to-high correlation coefficients.

**Table 3:** Spearman’s correlations between the OR4KT dimensions (*n* = 127)

	Climate for change	Contextual factors	Change content	Leadership	Organizational support	Motivation
Climate for change	1					
Contextual factors	.75	1				
Change content	.62	.67	1			
Leadership	.70	.64	.55	1		
Organizational support	.55	.62	.53	.70	1	
Motivation	.45	.60	.46	.53	.45	1

#### Concurrent validity: relations with external variables

We first verified the concurrent validity of the dimensions by investigating whether the OR4KT score was affected by the characteristics of primary care units or the sociodemographic characteristics of key informants. No significant associations were detected for any of these characteristics, and therefore we did not perform stratified analyses.

Second, we calculated the intra-center correlation coefficient (ICC) for each dimension and for the total summary score in the OR4KT and SOAPC. Since the agreement is acceptable (for example 0.244 for the total score), we consider the aggregation of scores to be justified for obtaining a collective measure of readiness for change by health center. Then, we assessed whether the mean score obtained by primary care units for the six dimensions and the total score of the OR4KT were associated with their score for the four dimensions and the total score of the SOAPC, using Spearman’s correlation. Results are shown in Table 4.

**Table 4:** Spearman’s correlations between dimensions from the OR4KT and the SOAPC

	SOAPC				
OR4KT	Communication	Decision making	Change history	Stress	Total score
Climate for change	.78*	.76*	− .3	.62*	.82*
Contextual factors	.81*	.80*	− .1	.46*	.80*
Change content	.71*	.64*	.11	.61*	.80*
Leadership	.58*	.68*	.10	.32	.62*
Organizational support	.65*	.80*	.05	.26	.64*
Motivation	.34	.39	.08	.19	.40
Total score	.74*	.74*	.02	.49*	.76*

*significant correlation (*p* < .05)

In general, correlations between SOAPC and OR4KT scores were positive and moderate. The most notable exception is the history of change dimension from the SOAPC that shows no significant association with any of the OR4KT dimensions, and this supports the concurrent validity of the questionnaire. In fact, this dimension mostly reflects past experience with change whereas the OR4KT assesses the readiness to implement change in the future. In addition, the stress dimension of the SOAPC is not significantly correlated with several OR4KT dimensions, namely leadership, organizational support, and motivation.

**Fig. 2:**
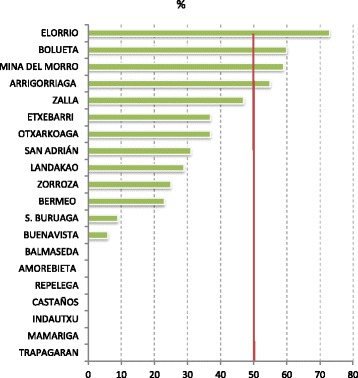
Level of commitment in participating primary care units

Third, we analyzed the relationship between the total OR4KT score and the level of commitment in each primary care unit as reflected by the percentage of professionals who signed consent forms agreeing to collaborate in the PVS-III project (see Fig. 2). Among the 20 participating primary care units, seven were given a score of zero with respect to their level of commitment for the following reasons: (1) the head of the unit changed over the course of the study; (2) the selection of key informants did not follow the required process; (3) the project presentation meeting did not take place; or (4) the decision regarding participation in the PVS-III project was not individual but collective (i.e., if a primary care unit decided to participate, all members staff of this unit were required to participate).

The Spearman’s rank correlation between the total OR4KT score and the level of commitment was .80 (*p* < .001) when eliminating the six primary care units with a score of zero and .67 (*p* < .001) if we consider all 20 participating primary care units.

#### Known-groups validity

To test known-groups validity, we performed a ROC curve analysis to determine the OR4KT score level that better differentiated between primary care units with more and less than 50% of the staff having signed a collaboration consent form. As shown in Table 5, the best cut-off value, that is, the one that maximizes sensitivity and specificity, is a score of 64.48 points (out of 100) on the OR4KT total score.

**Table 5:** Sensitivity and (1-specificity) for level of commitment for a range of OR4KT scores

Positive if OR4KT score ≥	Sensitivity	1-specificity
55.10	1.00	.89
56.97	1.00	.78
57.19	1.00	.67
58.25	1.00	.56
59.68	1.00	.44
60.85	1.00	.33
61.72	.75	.33
62.34	.75	.22
63.45	.75	.11
64.48	.75	.00
64.77	.50	.00
66.36	.25	.00
68.94	.00	.00

## Discussion

This study aimed to provide an initial assessment of the validity and reliability of the Spanish version of the OR4KT instrument in the context of the implementation of the PVS-III project, which seeks to strengthen health promotion and chronic disease prevention in primary healthcare organizations of the Basque Health Service. Based on a collaborative approach and shared decision making between primary care providers and community members, the PVS-III model requires a high level of intra- and inter-organizational cooperation as well as the involvement of multiple sectors [5–7]. Given this, it was deemed essential to assess the readiness of primary healthcare organizations to implement this approach, to enable us to carefully select centers with the highest motivation for adopting the proposed change.

The results of the initial validation process show that the Spanish OR4KT questionnaire is a reliable instrument, based on the assessment of the internal consistency and repeatability. The Cronbach’s alpha for the overall OR4KT instrument is .95, and the overall CR is 6.92%, statistics which provide strong support for the reliability of this instrument.

Compared to other available OR assessment instruments (e.g., [8, 14, 23]), the internal consistency of the OR4KT is high, with four of the six dimensions showing Cronbach’s alphas of .80 and higher, while the two other dimensions—climate for change and contextual factors—had alpha coefficients of .79. Initial checks showed that removing some items from these two dimensions would increase these coefficients to above .80. Nevertheless, we decided to maintain all the original items from the OR4KT in the factor analysis model given that it was the first attempt to validate this instrument in a specific implementation project.

The overall repeatability score of the OR4KT was highly satisfactory and satisfactory for the six dimensions. A CR < 10% is considered to indicate excellent repeatability, and a CR between 11 and 20% is considered adequate [26]. It is worth noting that very few OR instruments have reported repeatability to date [17], and in this sense, our study contributes to knowledge of the reliability of OR measures.

The validity of the OR4KT was assessed by three methods, considering internal as well as external evidence. First, we performed CFAs to provide internal structure validity evidence by assessing the degree to which individual items fit the underlying construct of interest. The goal of this structure was to integrate the configuration of the items by combining six first order factors with one second order factor, the OR4KT total score. Initially, CFA on the item parcels created by the sub-dimension composites was performed. Later, a second order structure beyond the sub-dimension composites was evaluated. Both models showed acceptable fit. The loads are greater than 0.40 for most items and sub-dimensions, except for items 8, 15, 29, 32, 50, and 52 with loads between .30 and .40. Given the limited sample size and the fact that this study was conducted in a specific implementation context, it is premature to suggest changes to the OR4KT structure and measurement items at this moment. Once the metric properties of the English and French versions are analyzed, the actions to be performed with the items that have less than optimal operation will be studied.

Second, we assessed the relationships between the six dimensions of the instrument. All correlations were positive and significant.

Third, to assess concurrent validity of the OR4KT questionnaire, we explored the association between the dimensions assessed by the OR4KT and those assessed by the SOAPC, using Spearman’s correlations. Significant correlations were found between dimensions that are similar in the two instruments but not between dimensions that are unique to one of them (e.g., no correlation was found between motivation in the OR4KT and history of change in the SOAPC). The fact that several dimensions from the OR4KT and the SOAPC are correlated suggests further exploring the relative contribution of these instruments to assess healthcare organizations’ readiness to implement evidence-informed change. The SOAPC has been developed specifically for primary care organizations whereas the OR4KT could be used across a wide range of healthcare organizations.

Also, we tested the known-groups validity of the OR4KT using ROC curve analysis in order to determine the OR4KT score that best differentiated centers with a high level of commitment (i.e., those where at least 50% of the professionals signed individual consent forms agreeing to participate in the PVS-III project). The results indicate that a score of 64.48 (out of 100) on the OR4KT was the optimal cut-off point for this purpose. That is, an OR4KT score above this value would be indicative of an organization with good readiness for implementing the intervention under study.

### Strengths and limitations

This study reports the first validation and application of the Spanish version of the OR4KT instrument that assesses an organization’s level of readiness to implement evidence-informed practices. The OR4KT may be useful as a screening tool to select organizations with strong potential for successful implementation of evidence-informed health promotion practices in primary care. Like other available OR instruments, such as the IEMAC [14] and the DCCRA [15], the OR4KT could also be used to evaluate the implementation of new practices and to follow changes over time, or as a monitoring tool for quality improvement. However, these applications have not yet been tested for the OR4KT.

This initial validation of the OR4KT questionnaire was conducted alongside the real-life implementation of the PVS-III project to improve health promotion practices in primary care. This pragmatic approach has allowed us to collect useful data to inform future implementation strategies of the PVS-III project, which constitutes a major strength of this study. However, the study has some limitations. First, the number of eligible primary care centers was limited to 49, and the final response rate was 40%, thereby providing a sample of 20 organizations. Nevertheless, the number of individual respondents from participating organizations that completed the test (127) and retest (90) surveys can be considered acceptable.

Second, the OR4KT is built as a combination of measures from several OR instruments. Given our choice to keep the OR4KT questionnaire convenient for busy healthcare providers and managers, item reduction was performed, to minimize the number of questions and hence the time required to complete the assessment. However, it is possible that the deletion of some items has modified the structure of the constructs.

In addition, the results obtained from the ROC analysis should be taken with caution for 5 reasons: (1) the sample size is too small, as so few of the centers seemed to be ready in the first place; (2) we consider that those centers in which the majority of the professionals commit to participate and sign collaboration consent are clearly different from those in which those professionals are in minority. Absolute majority is the most popular method to make group decisions and reach group consensus, which are the fundamental procedure under the process of collaborative modeling of PVS programs, but it is an experiential cut-off. (3) The scores from all facilities ranged from 52.10 to 68.94; (4) three of the facilities where less than 50% of physicians signed up had a higher score than a facility where > 50% of physicians signed up; and (5) the difference between the cut-off score and the next lowest score (which was achieved by a facility considered as “disengaged”) is small (63.45 vs. 64.48).

### Future research

We consider that factorial invariance is a desirable property of a measurement instrument, so we expect to perform a progressive invariance analysis between versions (configural invariance, metric invariance, and strict factor invariance) once we have the necessary data for the analysis of the metric properties of each version of the questionnaire.

We also aim to carry out additional research to verify if the adaptation of the OR4KT items to the specific context, scope, and type of intervention to be implemented (e.g., the PVS-III program) produces changes in the metric properties of the instrument with respect to the current version of the questionnaire, in which generic items were used alongside an introduction in the instructions on the context of applying the intervention.

Finally, it would be convenient to carry out more analysis of known groups once we know that health centers of participants in PVS-III have reached the standards to consider that they have modified their preventive practice.

## Conclusion

Primary healthcare offers invaluable opportunities for promoting a healthy lifestyle, but practices remain suboptimal. Program implementation should be guided by a careful examination of the factors that could enable or hinder the integration of proposed changes. The OR4KT is a 59-item questionnaire available in three languages that could help in the selection of organizations that are most likely to successfully implement change interventions. The Spanish version of the OR4KT questionnaire that was initially validated in this study exhibits very good psychometric properties, although it needs to be validated in a larger sample and in other implementation contexts.

## Additional files

Additional file 1: Spanish version of the OR4KT questionnaire. (PDF 1029 kb)

Additional file 2: The OR4KT factorial configuration matrix. (DOCX 18 kb)

## Abbreviations

CFA: Confirmatory factor analysis
CFI: Comparative Fit Index
CR: Coefficient of Repeatability
DCCRA: Diabetes Care Coordination Readiness Assessment
ICC: Intra-class correlation coefficient
IEMAC: Instrumento de Evaluación de Modelos de Atención ante la Cronicidad
Kurt.: Kurtosis
M: Mean
Max.: Maximum score
Min.: Minimum score
MLMV: Maximum likelihood mean-variance
OR: Organizational readiness
OR4KT: Organizational Readiness for Knowledge Translation
ORIC: Organizational Readiness for Implementing Change
PVS-III: Prescribe Vida Saludable III
ROC: Receiver operating characteristic curve
SD: Standard deviation
Sk: Skewness
SOAPC: Survey of Organizational Attributes for Primary Care
SRMR: Standardized Root Mean Square Residual
