# Supplementary material for: Validity and reliability of the Spanish version of the Organizational Readiness for Knowledge Translation (OR4KT) questionnaire
Source: Implement Sci. 2017 Nov 10;12:128. doi: 10.1186/s13012-017-0664-y (PMC5681775; doi:10.1186/s13012-017-0664-y)
Supplement: Supplementary file 1 — Spanish version of the OR4KT questionnaire. (PDF 1029 kb) [file 13012_2017_664_MOESM1_ESM.pdf]

Predisposición organizacional a la transferencia del conocimiento para el cambio de práctica clínica

¡¡Recuerda!! Para contestar a este cuestionario debes pensar en el cambio, intervención, nuevas prácticas etc. en el contexto de la innovación para promover hábitos de vida saludables (actividad física, abandono del tabaco, consumo moderado de alcohol y dieta equilibrada).

Utilizando esta escala de cinco puntos, valora tu centro de salud en relación con cada una de las afirmaciones siguientes:

1 = Totalmente en desacuerdo; 2 = En desacuerdo; 3 = Neutro; 4 = De acuerdo; 5 = Totalmente de acuerdo

**1. Clima organizacional para el cambio**					
**En tu centro de salud**	**1**	**2**	**3**	**4**	**5**
1. los profesionales trabajan en equipo	☐	☐	☐	☐	☐
2. los profesionales están habitualmente pendientes de ayudarse entre si cuando se necesita	☐	☐	☐	☐	☐
3. la confianza mutua entre los profesionales es fuerte	☐	☐	☐	☐	☐
4. la sobrecarga de trabajo reduce la efectividad de las intervenciones	☐	☐	☐	☐	☐
5. la frustración de los profesionales es frecuente	☐	☐	☐	☐	☐
6. las ideas y sugerencias de los profesionales reciben la debida consideración por parte de la dirección	☐	☐	☐	☐	☐
7. los canales de comunicación formales funcionan muy bien	☐	☐	☐	☐	☐
8. los profesionales se sienten libres para formular preguntas y expresar sus preocupaciones	☐	☐	☐	☐	☐
9. la dirección de comarca está abierta a las ideas de los profesionales para propiciar los cambios	☐	☐	☐	☐	☐
10. la dirección de comarca favorece prácticas nuevas y diferentes	☐	☐	☐	☐	☐

¡¡Recuerda!! Para contestar a este cuestionario debes pensar en el cambio, intervención, nuevas prácticas etc. en el contexto de la innovación para promover hábitos de vida saludables (aumento actividad física, abandono del tabaco, consumo moderado de alcohol y dieta equilibrada).

Utilizando esta escala de cinco puntos, valora tu centro de salud en relación con cada una de las afirmaciones siguientes:

1 = Totalmente en desacuerdo; 2 = En desacuerdo; 3 = Neutro; 4 = De acuerdo; 5 = Totalmente de acuerdo

**2. Factores contextuales y organizacionales**					
**Tu centro de salud**	**1**	**2**	**3**	**4**	**5**
11. tiene capacidad de determinar roles y responsabilidades para realizar un cambio en las prácticas preventivas	☐	☐	☐	☐	☐
12. tiene el apoyo necesario en términos de presupuesto o recursos económicos para realizar un cambio en las prácticas preventivas	☐	☐	☐	☐	☐
13. tiene el apoyo necesario en términos de formación	☐	☐	☐	☐	☐
14. tiene el apoyo necesario en términos de instalaciones y equipamiento	☐	☐	☐	☐	☐
15. tiene el apoyo necesario en cuanto a la dotación de personal	☐	☐	☐	☐	☐
**En tu centro de salud**	**1**	**2**	**3**	**4**	**5**
16. la dirección de comarca solicita las opiniones del personal clínico en la toma de decisiones sobre la atención al paciente	☐	☐	☐	☐	☐
17. los profesionales tienen sentido de responsabilidad profesional para mejorar la atención al paciente y los resultados	☐	☐	☐	☐	☐
18. los profesionales cooperan para mantener y mejorar la eficacia en la atención al paciente	☐	☐	☐	☐	☐
19. los profesionales están dispuestos a innovar y/o experimentar para mejorar los procedimientos clínicos	☐	☐	☐	☐	☐
20. los profesionales son receptivos al cambio en los procesos clínicos	☐	☐	☐	☐	☐

¡¡Recuerda!! Para contestar a este cuestionario debes pensar en el cambio, intervención, nuevas prácticas etc. en el contexto de la innovación para promover hábitos de vida saludables (aumento actividad física, abandono del tabaco, consumo moderado de alcohol y dieta equilibrada).

Utilizando esta escala de cinco puntos, valora tu centro de salud en relación con cada una de las afirmaciones siguientes:

1 = Totalmente en desacuerdo; 2 = En desacuerdo; 3 = Neutro; 4 = De acuerdo; 5 = Totalmente de acuerdo

1 = Totalmente en desacuerdo; 2 = En desacuerdo; 3 = Neutro; 4 = De acuerdo; 5 = Totalmente de acuerdo

3. Contenido del cambio

En tu centro de salud	1	2	3	4	5
21. existe voluntad para ajustarse a los cambios	☐	☐	☐	☐	☐
22. existe la capacidad para intercambiar ideas y tener impacto sobre las decisiones vinculadas a la atención al paciente	☐	☐	☐	☐	☐
23. existe la flexibilidad necesaria para adaptarse al cambio	☐	☐	☐	☐	☐
24. los profesionales están dispuestos a hacer ajustes en el trabajo habitual en respuesta a lo que está sucediendo a su alrededor	☐	☐	☐	☐	☐
25. generalmente, se pueden adaptar nuevas normas o procedimientos al trabajo habitual, incluso los que son impuestos	☐	☐	☐	☐	☐
26. generalmente, los cambios propuestos han sido bien recibidos por parte de los pacientes	☐	☐	☐	☐	☐
27. los cambios propuestos toman en consideración las necesidades y preferencias de los pacientes	☐	☐	☐	☐	☐
28. los cambios propuestos parecen tener más ventajas que desventajas para los pacientes	☐	☐	☐	☐	☐
29. los cambios propuestos deberían ser efectivos, basados en los conocimientos científicos actuales	☐	☐	☐	☐	☐

¡¡Recuerda!! Para contestar a este cuestionario debes pensar en el cambio, intervención, nuevas prácticas etc. en el contexto de la innovación para promover hábitos de vida saludables (aumento actividad física, abandono del tabaco, consumo moderado de alcohol y dieta equilibrada).

Utilizando esta escala de cinco puntos, valora tu centro de salud en relación con cada una de las afirmaciones siguientes:

1 = Totalmente en desacuerdo; 2 = En desacuerdo; 3 = Neutro; 4 = De acuerdo; 5 = Totalmente de acuerdo

**4. Liderazgo**					
**En tu centro de salud**	**1**	**2**	**3**	**4**	**5**
30. la dirección de comarca ofrece una gestión eficaz para una mejora continua de la atención al paciente	☐	☐	☐	☐	☐
31. la dirección de comarca ofrece a los profesionales retroalimentación/datos sobre los efectos de las decisiones clínicas	☐	☐	☐	☐	☐
32. la dirección de comarca pide cuentas a los empleados de los resultados alcanzados	☐	☐	☐	☐	☐
33. los agentes externos participan en el proceso de planificación	☐	☐	☐	☐	☐
34. generalmente, todos los empleados están involucrados en los procesos de toma de decisiones.	☐	☐	☐	☐	☐
35. hay un responsable de las decisiones de innovación entre los clínicos	☐	☐	☐	☐	☐
36. hay un responsable de las decisiones de innovación entre los administrativos	☐	☐	☐	☐	☐
37. la dirección de comarca participa en los procesos de cambio	☐	☐	☐	☐	☐
38. los profesionales clínicos participan en los procesos de cambio	☐	☐	☐	☐	☐
39. el personal no clínico participa en los procesos de cambio	☐	☐	☐	☐	☐

¡¡Recuerda!! Para contestar a este cuestionario debes pensar en el cambio, intervención, nuevas prácticas etc. en el contexto de la innovación para promover hábitos de vida saludables (aumento actividad física, abandono del tabaco, consumo moderado de alcohol y dieta equilibrada).

Utilizando esta escala de cinco puntos, valora tu centro de salud en relación con cada una de las afirmaciones siguientes:

1 = Totalmente en desacuerdo; 2 = En desacuerdo; 3 = Neutro; 4 = De acuerdo; 5 = Totalmente de acuerdo

**5. Apoyo organizacional**					
**En tu centro de salud**	**1**	**2**	**3**	**4**	**5**
40. los miembros del equipo brindan apoyo real a las nuevas ideas y sus aplicaciones	☐	☐	☐	☐	☐
41. los miembros del equipo ponen fácil el desarrollo de nuevas ideas	☐	☐	☐	☐	☐
42. los miembros del equipo cooperan para ayudar a desarrollar y aplicar nuevas ideas	☐	☐	☐	☐	☐
43. entre los miembros del equipo se comparten recursos para facilitar la aplicación de nuevas ideas	☐	☐	☐	☐	☐
44. los procesos de cambio son monitorizados continuamente	☐	☐	☐	☐	☐
45. los resultados son monitorizados continuamente	☐	☐	☐	☐	☐
46. la evaluación y la mejora de la implementación del cambio incluye mediciones periódicas de los resultados	☐	☐	☐	☐	☐
47. la evaluación y la mejora de la implementación del cambio incluye un plan de divulgación de los resultados	☐	☐	☐	☐	☐
48. la evaluación y la mejora de la implementación del cambio incluye la revisión de los resultados por los líderes	☐	☐	☐	☐	☐
49. hay mecanismos formales establecidos para recoger los comentarios relacionados con el cambio propuesto	☐	☐	☐	☐	☐

¡¡Recuerda!! Para contestar a este cuestionario debes pensar en el cambio, intervención, nuevas prácticas etc. en el contexto de la innovación para promover hábitos de vida saludables (aumento actividad física, abandono del tabaco, consumo moderado de alcohol y dieta equilibrada).

Utilizando esta escala de cinco puntos, valora tu centro de salud en relación con cada una de las afirmaciones siguientes:

1 = Totalmente en desacuerdo; 2 = En desacuerdo; 3 = Neutro; 4 = De acuerdo; 5 = Totalmente de acuerdo

**6. Motivación**					
**En tu centro de salud**	**1**	**2**	**3**	**4**	**5**
50. los pacientes presionan para que se realicen cambios en las prácticas preventivas	☐	☐	☐	☐	☐
51. las presiones para realizar cambios en las prácticas preventivas provienen de los profesionales	☐	☐	☐	☐	☐
52. la dirección de comarca presiona para realizar cambios en las prácticas preventivas	☐	☐	☐	☐	☐
53. las presiones para realizar cambios en las prácticas preventivas provienen de Osakidetza	☐	☐	☐	☐	☐
54. el Departamento de Salud presiona para realizar cambios en las prácticas preventivas	☐	☐	☐	☐	☐
55. existe experiencia previa de implementación de cambios en las prácticas preventivas, obtenida de proyectos o programas pilotos y sus evaluaciones	☐	☐	☐	☐	☐
56. los directivos conocen la innovación gracias a su experiencia previa	☐	☐	☐	☐	☐
57. se dispone de conocimientos sobre cómo están siendo utilizadas por otras organizaciones innovaciones similares en las prácticas preventivas	☐	☐	☐	☐	☐
58. los directivos promueven el cambio comportándose de manera consistente con dicho cambio en las prácticas preventivas	☐	☐	☐	☐	☐
59. los directivos definen el curso del cambio en las prácticas preventivas	☐	☐	☐	☐	☐

Comentarios:

☐
