# Supplementary material for: Validity and reliability of the Spanish version of the Organizational Readiness for Knowledge Translation (OR4KT) questionnaire
Source: Implement Sci. 2017 Nov 10;12:128. doi: 10.1186/s13012-017-0664-y (PMC5681775; doi:10.1186/s13012-017-0664-y)
Supplement: Supplementary file 2 — The OR4KT factorial configuration matrix. (DOCX 18 kb) [file 13012_2017_664_MOESM2_ESM.docx]

| Sub-dimension | Standardized Factor Loading |
| --- | --- |
| Staff Cohesion |  |
| OR4KT_1 | 0.870 |
| OR4KT_2 | 0.743 |
| OR4KT_3 | 0.745 |
| Staff work-related stress |  |
| OR4KT_4REC | 0.443 |
| OR4KT_5REC | 0.931 |
| Communication about change |  |
| OR4KT_6 | 0.767 |
| OR4KT_7 | 0.922 |
| OR4KT_8 | 0.351 |
| Manager´s openness to change |  |
| OR4KT_9 | 0.867 |
| OR4KT_10 | 0.792 |
| Human resources to support change |  |
| OR4KT_11 | 0.418 |
| OR4KT_15 | 0.326 |
| Material resources to support change |  |
| OR4KT_12 | 0.642 |
| OR4KT_13 | 0.818 |
| OR4KT_14 | 0.559 |
| Organizational culture |  |
| OR4KT_16 | 0.516 |
| OR4KT_17 | 0.529 |
| OR4KT_18 | 0.637 |
| OR4KT_19 | 0.820 |
| OR4KT_20 | 0.698 |
| Attributes of change |  |
| OR4KT_21 | 0.785 |
| OR4KT_22 | 0.777 |
| Perceived complexity of change |  |
| OR4KT_23 | 0.826 |
| OR4KT_24 | 0.795 |
| OR4KT_25 | 0.698 |
| Patient experiences and preferences |  |
| OR4KT_26 | 0.631 |
| OR4KT_27 | 0.729 |
| OR4KT_28 | 0.578 |
| Research evidence supporting change |  |
| OR4KT_29 | 1.000 |
| Leadership/champion |  |
| OR4KT_30 | 0.677 |
| OR4KT_31 | 0.550 |
| OR4KT_32 | 0.320 |
| Strategic planning process |  |
| OR4KT_33 | 1.000 |
| Decision-making process |  |
| OR4KT_34 | 0.790 |
| OR4KT_35 | 0.629 |
| OR4KT_36 | 0.694 |
| Adequate level of involvement |  |
| OR4KT_37 | 0.564 |
| OR4KT_38 | 0.707 |
| OR4KT_39 | 0.684 |
| Support climate |  |
| OR4KT_40 | 0.841 |
| OR4KT_41 | 0.899 |
| OR4KT_42 | 0.898 |
| OR4KT_43 | 0.826 |
| Monitoring |  |
| OR4KT_44 | 0.930 |
| OR4KT_45 | 0.949 |
| Evaluation process |  |
| OR4KT_46 | 0.897 |
| OR4KT_47 | 0.902 |
| OR4KT_48 | 0.877 |
| Feedback |  |
| OR4KT_49 | 1.000 |
| Pressure for change |  |
| OR4KT_50 | 0.312 |
| OR4KT_51 | 0.674 |
| OR4KT_52 | 0.358 |
| OR4KT_53 | 0.391 |
| OR4KT_54 | 0.518 |
| Training and educational needs |  |
| OR4KT_55 | 1.000 |
| Adequate knowledge and skills |  |
| OR4KT_56 | 0.742 |
| OR4KT_57 | 0.628 |
| Commitment |  |
| OR4KT_58 | 0.841 |
| OR4KT_59 | 0.864 |
